# MEASURING QUALITY OF LIFE IN OLDER ADULTS LIVING WITH T2DM: A COMPARISON WITH YOUNGER ADULTS USING 2015 BRFSS DATA

**DOI:** 10.1093/geroni/igz038.966

**Published:** 2019-11-08

**Authors:** Brittany Smalls, Myles Moody, Matthew Rutledge, Amy Cowley

**Affiliations:** University of Kentucky, Lexington, Kentucky, United States

## Abstract

Challenges due to burden of disease can affect adherence to self-care behaviors and optimal health outcomes in those living with T2DM. This study utilized state- and national-level data from the 2015 BRFSS to compare QoL measured by the prevalence of physical and mental burden days among older adults (OAs) compared to younger adults living with T2DM. The results of our analysis showed that OAs living in the US were significantly less likely to experience at least one mental burden day when compared to their younger counterparts (OR =0.61, 95% CI: 0.58, 0.64), while gender, education, race, BMI, and depression, CVD, or another chronic condition were significantly associated with the odds of experiencing at least one mental burden day. Whereas, in Kentucky OAs were less likely to experience at least one mental burden day when compared to their younger counterparts (OR= 0.48, 95% CI: 0.35, 0.66). Gender, education, BMI, and depression were significantly associated with the odds of experiencing at least one mental burden day or one physical burden day. The findings of this study suggests that the questions used by BRFSS to measure QoL may not be the most suitable for OAs who likely have different criteria for self-reported mental or physical burden days. When assessing QoL or burden of disease among the aging at a population level, considerable thought should be given into the questions asked and if they appropriately examine patient-level QoL in this population.

